# Early maternal care and amygdala habituation to emotional stimuli in adulthood

**DOI:** 10.1093/scan/nsab059

**Published:** 2021-05-08

**Authors:** Nathalie E Holz, Alexander Häge, Michael M Plichta, Regina Boecker-Schlier, Christine Jennen-Steinmetz, Sarah Baumeister, Andreas Meyer-Lindenberg, Manfred Laucht, Tobias Banaschewski, Daniel Brandeis

**Affiliations:** Department of Child and Adolescent Psychiatry and Psychotherapy, Central Institute of Mental Health, Mannheim, Baden-Württemberg 68159, Germany; Department of Child and Adolescent Psychiatry and Psychotherapy, Central Institute of Mental Health, Mannheim, Baden-Württemberg 68159, Germany; Department of Psychiatry and Psychotherapy, Central Institute of Mental Health, Mannheim, Baden-Württemberg 68159, Germany; Department of Psychiatry, Psychosomatic Medicine and Psychotherapy, Goethe-Universität Frankfurt am Main, Frankfurt am Main, Hessen 60528, Germany; Department of Child and Adolescent Psychiatry and Psychotherapy, Central Institute of Mental Health, Mannheim, Baden-Württemberg 68159, Germany; Department of Biostatistics, Central Institute of Mental Health, Medical Faculty Mannheim, Heidelberg University, Mannheim, Baden-Württemberg 68159, Germany; Department of Child and Adolescent Psychiatry and Psychotherapy, Central Institute of Mental Health, Mannheim, Baden-Württemberg 68159, Germany; Department of Psychiatry and Psychotherapy, Central Institute of Mental Health, Mannheim, Baden-Württemberg 68159, Germany; Department of Child and Adolescent Psychiatry and Psychotherapy, Central Institute of Mental Health, Mannheim, Baden-Württemberg 68159, Germany; Department of Child and Adolescent Psychiatry and Psychotherapy, Psychiatric Hospital, University of Zurich, Zurich 8032, Switzerland; Department of Child and Adolescent Psychiatry and Psychotherapy, Central Institute of Mental Health, Mannheim, Baden-Württemberg 68159, Germany; Department of Psychiatry and Psychotherapy, Central Institute of Mental Health, Mannheim, Baden-Württemberg 68159, Germany; Department of Psychiatry, Psychosomatic Medicine and Psychotherapy, Goethe-Universität Frankfurt am Main, Frankfurt am Main, Hessen 6052; Department of Child and Adolescent Psychiatry and Psychotherapy, Central Institute of Mental Health, Mannheim, Baden-Württemberg 68159, Germany; Department of Child and Adolescent Psychiatry and Psychotherapy, Psychiatric Hospital, University of Zurich, Zurich 8032, Switzerland; Center for Integrative Human Physiology, Zurich 8057, Switzerland; Neuroscience Center Zurich, University of Zurich and ETH Zurich, Zurich 8057, Switzerland

**Keywords:** amygdala, maternal care, fMRI, resilience, habituation

## Abstract

Evidence suggests that maternal care constitutes a protective factor for psychopathology which may be conditional on the level of family adversity. Given that psychopathology is frequently linked with social deficits and the amygdala with social functioning, we investigated the impact of early maternal care on amygdala function under high *vs* low familial risk for psychopathology. Amygdala activity and habituation during an emotional face-matching paradigm was analyzed in participants of an epidemiological cohort study followed since birth (*n* = 172, 25 years). Early mother–infant interaction was assessed during a standardized nursing and play setting at the age of 3 months. Information on familial risk during the offspring’s childhood and on the participants’ lifetime psychopathology was obtained with diagnostic interviews. An interaction between maternal stimulation and familial risk was found on amygdala habituation but not on activation, with higher maternal stimulation predicting stronger amygdala habituation in the familial risk group only. Furthermore, amygdala habituation correlated inversely with Attention Deficit Hyperactivity Disorder (ADHD) diagnoses. The findings underline the long-term importance of early maternal care on the offspring’s socioemotional neurodevelopment and of interventions targeting maternal sensitivity early in life, particularly by increasing maternal interactive behavior in those with familial risk.

## Introduction

The level of early maternal care may mitigate the offspring’s risk for psychopathology. Evidence has suggested that this relation may be conditional on the level of family adversity (Woodward *et al.*, 2018) in line with the assumption that resilience occurs when risk is mitigated by a protective factor under adverse conditions (‘protective factor model’; Garmezy *et al.*, 1984). Hence, early maternal care might have a differential effect depending on the level of family risk, i.e. under high-risk conditions, such as parental psychopathology and low socioeconomic status, high levels of parental care may protect against effects of adversity (Egeland *et al.*, 1993; Pettit *et al.*, 1997; Wakschlag and Hans, 1999; Woodward *et al.*, 2018), while the impact in low-risk families may be less crucial. Accordingly, this pattern was demonstrated in young adults who were followed since birth in the context of the Mannheim Study of Children at Risk (Laucht *et al.*, 2000). Specifically, individuals with a familial risk for psychopathology had fewer ADHD diagnoses and were more sensitive to rewards during anticipation and less sensitive during delivery, provided that a high quality of early maternal care was present, leading to the proposal of a possible neural mechanism regarding how a protective environment may foster resilience under adverse circumstances (Holz *et al.*, 2018a). Given that most psychopathologies are associated with social deficits (Uekermann *et al.*, 2010), for which amygdala function may play a fundamental role (Sonuga-Barke *et al.*, 2016), and that parenting is a significant predictor of the offspring’s social and emotional skills (Cabrera *et al.*, 2007), affective processing is a particularly relevant phenotype to study. Therefore, we investigated the impact of early maternal care on the offspring’s amygdala function during emotional processing in individuals with a high *vs* low familial risk for psychopathology. Parental psychopathology is a major family adversity risk factor given that children of parents with mental health problems are at a tremendously increased risk of developing mental disorders themselves (Van Santvoort *et al.*, 2015b), thereby constituting the most prominent risk factor for psychopathology. Notably, studying children at high risk may provide important intervention implications, as these children can be easily identified by clinicians treating parent patients with mental diseases. However, effective early primary preventions for these high-risk children are still needed.

Aberrant amygdala function has been associated with a broad range of psychiatric disorders, ranging from internalizing to externalizing disorders, the latter including ADHD (Sonuga-Barke *et al.*, 2016). In terms of vulnerability to environmental adversity, a large number of studies have focused on the detrimental effects of severe forms of emotional stress on amygdala activation (Holz *et al.*, 2018b). In addition, evidence suggests that maternal psychopathology, such as prenatal maternal depression, might affect amygdala activity through alterations in its connectivity with the prefrontal cortex (Posner *et al.*, 2016). However, there is also evidence that this susceptibility to environmental influences may result in a positive outcome. In terms of amygdala morphometry, resilient animals showed decreased amygdala volume following a stress period (Anacker *et al.*, 2016). Moreover, positive parenting has been related to an attenuated growth during adolescence, which, in turn, has been associated with less psychopathology (Whittle *et al.*, 2013, 2014). In the same vein, it was highlighted that caregiver support buffers threat processing in the amygdala (for a review, see McLaughlin and Lambert, 2017). Consequently, amygdala function may serve as a protective factor.

Besides amygdala activation, amygdala habituation has been shown to be an important mechanism for the understanding of psychopathology. Habituation is the response decrement over time when stimuli are presented repeatedly. It is a very basic form of learning and confers an evolutionary advantage by determining how flexibly we are able to adapt to situations, giving priority to novel and salient stimuli while filtering out irrelevant information. Furthermore, amygdala habituation is a more reliable fMRI phenotype than amygdala mean activation (Plichta *et al.*, 2014) and might therefore be worthy of consideration in developmental contexts. Interestingly, decreased amygdala habituation has been reported in individuals with high anxiety (Hare *et al.*, 2008), high risk for social anxiety disorder (Blackford *et al.*, 2013) and autism spectrum disorder (Kleinhans *et al.*, 2009; Swartz *et al.*, 2013; Wiggins *et al.*, 2014). So far, its relation to externalizing disorders remains unknown, although studies have already highlighted the role of amygdala function in these disorders including ADHD (Plichta *et al.*, 2009; Tajima-Pozo *et al.*, 2015, 2018; Van Dessel *et al.*, 2019).

Likewise, research on environmental modifications of amygdala habituation is scarce, with one study finding that an interaction between a polymorphism in the endocannabinoid system and early adversity predicted amygdala habituation (Carey *et al.*, 2015) and another reporting increased habituation in patients with post-traumatic stress disorder (PTSD) who had been sexually abused (Van Den Bulk *et al.*, 2016). Remarkably, the ability to flexibly adapt emotional responses has been linked to resilient functioning (Waugh *et al.*, 2008; Westphal *et al.*, 2010), indicating that higher levels of amygdala habituation may possibly represent a protective factor.

With the present study, we aim to extend the above-reported interaction between familial liability and early maternal care by investigating whether the latter additionally serves as a protective factor in terms of emotion processing. Specifically, in analogy to our previous study on the interaction between familial risk and early maternal care, amygdala habituation is expected to be more pronounced in those with high familial risk with high-quality levels of early maternal care. In a second, albeit exploratory step, the relation between amygdala habituation and psychopathology, specifically ADHD was investigated, given that the above-mentioned interaction pattern has been highlighted as impacting on ADHD (Holz *et al.*, 2018a) and that the amygdala may be critical in terms of emotional dysregulation as prominently observed in ADHD (Hoogman *et al.*, 2017; Franke *et al.*, 2018). In line with clinical studies showing decreased habituation in various psychiatric diseases, it is hypothesized that amygdala habituation might act as a further protective factor in terms of ADHD, with more habituation being related to less lifetime ADHD. Such results would be particularly relevant as ADHD represents the most common neurodevelopmental disorder frequently accompanied by a range of coexisting internalizing symptoms (Polanczyk *et al.*, 2007; Taurines *et al.*, 2010; Franke *et al.*, 2018). Our findings could help to understand underlying protective mechanisms and to develop effective programs to promote resilience and thereby reduce the risk of developing psychiatric symptoms in high-risk children.

## Materials and methods

More details of the methods are provided in the Supplementary data.

### Sample

This investigation was conducted in the framework of the Mannheim Study of Children at Risk, an ongoing epidemiological cohort study of the long-term outcome of early risk factors (Laucht *et al.*, 2000). Out of 309 participants (80% of the original sample), only currently healthy participants were included in the neuroimaging sample (*n* = 172 for this investigation) in order to avoid confounding by current impairment. The study was approved by the ethics committee of the University of Heidelberg, and written informed consent was obtained from all participants.

### Assessments

#### Familial risk (parental psychiatric diagnoses).

As described in (Holz *et al.* 2018a), the presence of psychiatric diagnoses (personality, affective, substance-related and addictive, somatoform and stress disorders) in biological parents until the participants’ age of 11 years was assessed using diagnostic interviews with the parents [Mannheim Parent Interview (Esser *et al.*, 1989)]. The interview was conducted by informed trained psychologists at each of the five assessments during childhood, yielding a dichotomous variable (0 = not present and 1 = present).

#### Early mother–child interaction.

As described in Holz *et al.* (2018a), videotapes of a 10 min standardized nursing and play situation between mothers and their 3-month-olds at our lab were recorded and evaluated by trained raters (κ > 0.83) using a modified version of the category system for micro-analysis of the early mother–child interaction (Jörg *et al.*, 1994; Schmid *et al.*, 2011). Raters were blind to parental and child risk status. Nine measures of mother–infant interaction behavior were formed by coding a behavior as present or absent in a total of 120, 5 s intervals. Maternal stimulation included all attempts to attract the infant´s attention or to establish contact with him/her (vocal, facial or motor) and was coded when the baby was gazing at the mother or when the behaviors were clearly directed at the child. Maternal responsiveness comprised all behaviors executed in response to the infant behaviors (vocal, facial or motor). Infant responsiveness was assessed accordingly and added as a covariate in all interaction models, including maternal stimulation and responsiveness, to ensure that the effects were specifically attributable to maternal behavior. With this exception, all z-transformed measures were considered separately in all analyses.

#### Psychosocial adversity.

Information on adverse characteristics of the parents (low educational level, broken home history or delinquency and poor coping skills), their partnership (early parenthood, one-parent family, unwanted pregnancy and marital discord) and the family environment (overcrowding, poor social integration and support, and severe chronic life difficulties) was assessed according to an ‘enriched’ family adversity index (Holz *et al.*, 2016) by a standardized parent interview conducted at each assessment until the age of 11 years (range 0–9, *M* = 2.95; s.d. = 2.05). In this report, a modified version (excluding parental psychiatric diagnoses) was used as compared to, e.g. Holz *et al.* (2016).

#### Life events.

To assess exposure to life stress (LS) after the age of 11 years, a semi-structured parent interview was conducted at the age of 15 years. The young adults were interviewed from the age of 19 years onwards. The interview, which was a modified and shortened version of the Munich Events List (Maier-Diewald *et al.*, 1983), evaluated the occurrence of adverse life events during a period of 1 year prior to the assessment. The items (42–59, depending on developmental period) covered all relevant areas of children’s and young adults’ LS, including family, school, parents, health, legal troubles, and living conditions, such as birth of a sibling, death of a close relative or parents’ separation. A composite score was computed by summing up the z-standardized scores from the five assessments between the age of 15 and 25 years.

#### Child and adolescent psychopathology.

Sum scores for the presence of ADHD diagnoses (lifetime ADHD), disruptive symptoms/conduct disorder (CD) diagnoses and mood/anxiety diagnoses during childhood and adolescence were assessed using diagnostic interviews with the parents (Esser *et al.*, 1989) until age of 11 years and with the children at ages of 8 and 11 years. At age of 15 years, the Schedule for Affective Disorders and Schizophrenia for School-Age Children [K-SADS-PL (Delmo *et al.*, 2000)] was conducted independently with parents and adolescents and, at the age of 19 years, the Structured Clinical Interview for Diagnostic and Statistical Manual of Mental Disorders (DSM)-IV (Wittchen *et al.*, 1997) was performed with the offspring. A diagnosis was defined as present when criteria were met in either the parent or adolescent interview. The presence of a diagnosis (0 = not present and 1 = present) for each assessment (*n* = 6) was then added up to a sum score.

#### Substance abuse.

The results were additionally controlled for lifetime substance abuse, including lifetime nicotine dependence [Fagerström Test for Nicotine Dependence (FTND) (Heatherton *et al.*, 1991)], lifetime alcohol abuse [Alcohol Use Disorders Identification Test (AUDIT) (Babor *et al.*, 2001)] and lifetime cannabis abuse (12- month prevalence).

### Faces t*a*sk

To assess emotion processing, the participants were presented with 12 blocks of sequences of fearful/angry faces alternated with sequences of shapes (Hariri *et al.*, 2002). In the face blocks, participants were instructed to indicate which of the two faces at the bottom was identical to the target face (at the top) and to press the button on the respective side. In the sensorimotor control task, participants had to compare circles and ellipses according to the same criterion. Both stimulus sets consisted of six different trios of faces, one of which consisted of both, fearful and angry faces or shapes, each presented every 2.5 s on average.

### Data analysis

Functional magnetic resonance imaging was performed at the age of 25 years using a 3 Tesla scanner (Magnetom TRIO, Siemens, Erlangen, Germany) with a standard 12-channel head coil. Functional images were analyzed using Statistical Parametric Mapping (SPM8, http://www.fil.ion.ucl.ac.uk/spm) implemented in Matlab 7.12. (Mathworks Inc., Natick, MA, USA) with standard preprocessing steps.

Habituation-specific analyses were conducted according to Plichta *et al.* (2014). First-level temporal modeling within a general linear model (GLM) framework was performed to generate 3D maps of estimated regressor response amplitudes. The design matrices included 12 block regressors (6 faces and 6 shapes), which were convolved with the default SPM hemodynamic response function. Motion parameters were included as additional covariates of no interest, and contrast images were visually checked for possible residual movement. A high-pass filter with a cut-off frequency of 1/359 Hz was used to attenuate only the lowest frequency components (linear scanner drifts). All analyses were corrected for serially correlated errors by fitting a first-order autoregressive process (AR[1]) to the error term. The habituation of brain activity was calculated as linear within-subject contrast capturing the time effect across the difference images of all of six contrast blocks of faces *vs* shapes. The weighting corresponds to the linear contrast, usually given as an output by standard programs for repeated measures ANOVAs. General task effects were obtained using a one sample *t*-test of each of these three contrast images with whole-brain Family-Wise Error (FWE) correction at *P* < 0.05. An additional control for absolute translation and rotation movement in the second-level analysis did not change the results suggesting that habituation was not confounded by head motion. Then, the linear contrast images were entered into second-level group multiple regression analyses (separate regressions for each measure of mother‒child interaction), with the interaction term between parental psychiatric diagnoses and maternal stimulation or maternal or infant responsiveness, as the main predictor while all main effects and sex were entered as additional covariates. Infant responsiveness was additionally controlled for in the models including maternal measures, i.e. maternal stimulation by familial risk and maternal responsiveness by familial risk, to ensure that the effects could be solely attributed to maternal behavior. However, the results did not differ when this additional covariate was not included. Based on our previous results (Holz *et al.*, 2018a), we hypothesized that only the interaction between maternal stimulation and familial risk would have an impact on the amygdala habituation. Separate Region of Interest (ROI) masks for the left and right amygdalae were defined from the WFU-PickAtlas (Maldjian *et al.*, 2003), where a *P* < 0.05 FWE correction (minimum of five adjacent voxels) was applied. Exploratory whole-brain analyses of the interaction effect were performed at *P*_FWE_ < 0.05. With a conservative Bonferroni correction for the four interaction models tested (two mother–child interaction measures and two hemispheres), the results would still reach significance (*P* = 0.01). Moreover, several sensitivity analyses additionally controlling for environmental adversity, mood and anxiety disorders, and lifetime internalizing diagnoses in the offspring, which themselves have also been related to aberrant amygdala habituation, were performed to reveal the robustness of the interaction effect. Given the nature of the caretaker analyses, we further examined whether our effects were specific for maternal or paternal psychiatric diagnoses, using the interaction between these and maternal stimulation separately as a predictor in all regression analyses. Furthermore, the main interactions were additionally investigated following the recommendation by Keller (2014), i.e. including all predictor by covariate interactions (see also Supplementary Table S2 for an overview of the regression models). Mean contrast values of each participant were extracted from the significant cluster and exported to SPSS Statistics 20 (IBM, Armonk, NY), enabling visualization (a negative slope means increased habituation). As all plots were adjusted for covariates, negative values can emerge. In addition, the association between lifetime ADHD (predictor) and the contrast values was calculated by means of regression analyses controlling for sex in SPSS, with a significance threshold of *P* < 0.05 considered as statistically significant. In a further step, outliers with leverage values >0.2 (Huber, 1981) and cook’s distance >1 were excluded to ensure that the strength of the regression relationship was not inflated. Mediation analysis using the Sobel test (Baron and Kenny, 1986) was applied in the two groups defined by the presence of familial risk, with the assumption that externalizing diagnoses were the mediator of the relationship between maternal stimulation and amygdala habituation.

Regarding behavioral performance, habituation over time was tested for reaction time and accuracy using a repeated measure ANOVA on difference scores for the face *vs* the shape condition.

## Results

### Sample characteristics

Individuals with high familial risk received less maternal stimulation early in life (*t*(170) = 3.17 and *P* = 0.002) across all percentile ranges (details in Supplementary Table S1) and had more lifetime psychiatric diagnoses (ADHD: *t*(170) = −2.24, *P* = 0.03; CD diagnoses (*t*(170) = −2.86, *P* = 0.005; internalizing: *t*(170) = −2.12, *P* = 0.03) when compared to the low-risk group. Moreover, higher maternal stimulation but not maternal responsiveness predicted less lifetime ADHD (β = −18, *t*(169) = −2.68, *P* = 0.008), but did not predict either CD (*P* = 0.13) or internalizing diagnoses (*P* = 0.99). ADHD correlated with CD but was unrelated to lifetime depression (*r* = 0.11, *P* = 0.12) and anxiety (*r* = −08, *P* = 0.33). Details are depicted in Table 1.

**Table 1.** Sample characteristics by presence of parental psychiatric disorder during childhood

Parental psychiatric diagnosis	Not present	Present	Test statistics	*P*-value
*n* (%)	89 (51.7)	83 (48.3)		
Males, *n* (%)	35 (39.33)	37 (44.58)	Χ^2^(1) = .49	0.48
Maternal stimulation, mean (SD)^a^	0.23 (0.95)	−0.24 (1.00)	*T*(170) = 3.17, *d* = 0.47^b^	0.002
Maternal responsiveness, mean (SD)^a^	−0.06 (0.98)	0.08 (1.01)	*T*(170) = −.90	0.37
Infant responsiveness, mean (SD)^a^	−0.04 (0.94)	0.06 (1.05)	*T*(170) = −.68	0.50
ADHD diagnosis, *n* (%)	17 (19.1)	25 (30.12)	Χ^2^(1) = 2.83	0.09
Sum of ADHD diagnoses, mean (SD)	0.27 (0.62)	0.58 (1.11)	*T*(170) = −2.24, *d* = −0.35^b^	0.03
Disruptive behaviors and CD diagnoses, *n* (%)	10 (11.24)	22 (26.51)	Χ^2^(1) = 6.61, *V* = 0.20 ^c^	0.01
Sum of disruptive behaviors and CD diagnoses, mean (SD)	0.15 (0.47)	0.36 (0.85)	*T*(170) = −2.86, *d* = −0.43^b^	0.005
Mood and anxiety disorder, *n* (%)	19 (21.35)	24 (28.92)	Χ^2^(1) = 1.31	0.25
Mood and anxiety disorder, mean (SD)^a^	0.27 (0.58)	0.54 (1.03)	*T*(170) = −2.12, *d* = −0.17^b^	0.03

a *z*-transformed scores

b Cohen’s *d*

c Cramér’s *V*.

### Task effects of behavioral and neural habituation

Considering behavioral performance, a significant effect of time emerged with regard to reaction time for faces *vs* shapes (*F*(5,167) = 14.67, *P* < 0.001), indicating decreasing reaction times over the blocks. No effect emerged with regard to accuracy (*F*(5,167) = 0.52, *P* = 0.76), suggesting that the participants sustained their attention over time.

**Fig. 1.**
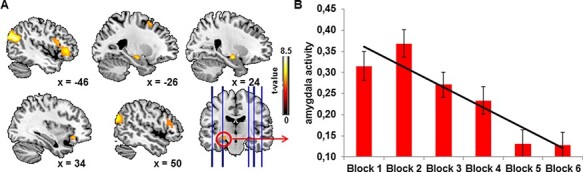
Linear habituation on whole brain level (*P*_FWE_  < 0.05) (A) and amygdala activation over the six blocks (B).

Whole-brain FWE-corrected analyses of the linear habituation contrasts revealed a response decrement in the left [*t*(171) = 6.43, *P*_FWE_ < 0.001] and right amygdala [*t*(171) = 6.66, *P*_FWE_ < 0.001]. Additional habituation was seen in the insula, the inferior frontal gyrus and several parts of the temporal and frontal gyri (Table 2, Figures 1 and S1).

**Table 2.** Whole brain FWE corrected linear habituation effect

Region	Brodman area	Cluster size	*t*-value	*P*(FWE)	Montreal Neurological Institute (MNI) coordinates		
					*x*	*y*	*z*
Middle temporal gyrus	39	876	8.37	<0.001	−50	−72	28
Middle temporal gyrus	39	408	6.93	<0.001	52	−72	26
Middle temporal gyrus	39		5.65	0.001	58	−60	28
Lateral Globus Pallidus		142	6.76	<0.001	26	−10	−10
Inferior frontal gyrus	46	527	6.50	<0.001	−46	34	2
Inferior frontal gyrus	44		6.32	<0.001	−52	18	12
Inferior frontal gyrus	9		5.08	0.011	−44	8	26
Amygdala		124	6.45	<0.001	−22	−12	−12
Inferior frontal gyrus	45	216	6.21	<0.001	58	24	14
Inferior frontal gyrus	46		5.37	0.003	54	32	8
Middle temporal gyrus	21	65	5.81	<0.001	−56	−6	−16
Insula	13	51	5.80	0.001	34	28	−10
Middle frontal gyrus	6	146	5.52	0.002	−30	10	58
Culmen		12	5.08	0.011	0	−36	−4
Sup. temporal gyrus	22	3	4.89	0.023	60	−4	−12
Thalamus		2	4.82	0.030	6	−6	0
Middle frontal gyrus	6	3	4.81	0.031	−38	0	46
Inferior frontal gyrus	47	10	4.79	0.034	−36	26	−14
Middle frontal gyrus	6	4	4.77	0.036	−38	6	50
Sup. frontal gyrus	6	3	4.77	0.036	−8	18	58

### Main effects

Amygdala habituation was not predicted by familial risk, maternal stimulation or infant responsiveness (all p_uncorrected_ > 0.009). However, maternal responsiveness was inversely related to the steepness of the slope of amygdala habituation [*t*(169) = 3.25, *P*_FWE_ = 0.01, R^2^ change = 0.06], resulting in less activity decrement over time in the offspring.

### Interaction effect

**Fig. 2.**
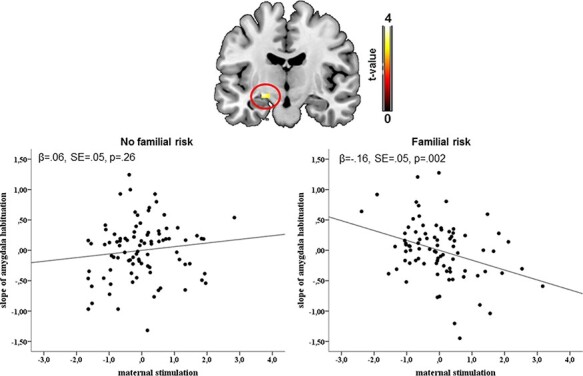
Maternal stimulation × familial risk interaction on amygdala habituation. Higher maternal stimulation was associated with a steeper slope of amygdala habituation, i.e. more amygdala habituation over time, in those participants with a high familial risk while maternal stimulation had no effect in the offspring with low familial risk.

An interaction effect of familial risk with maternal stimulation on left amygdala habituation was obtained (*t*(166) = 3.87, *P*_FWE_ = 0.003, R^2^ change = 0.06; Figure 2), while the impact on right amygdala habituation fell short of significance (*t*(166) = 2.65, *P*_FWE_ = 0.06). In detail, higher maternal stimulation was associated with a steeper slope of amygdala habituation, i.e. more response decrement over time, in individuals with high familial risk (β = −.16, SE = 0.05, *P* = 0.002, R^2^ change = 0.13), while no significant relationship emerged in participants with low risk (β = 0.06, *P* = 0.26). The interaction remained significant when adjusted for initial amygdala activity in the first block [*t*(165) = 3.89, *P*_FWE_ = 0.003] and also when all covariate by predictor interactions were included [*t*(162) = 3.82, *P*_FWE_ = 0.004]. On a whole-brain level, no region survived whole-brain FWE correction. Moreover, no interaction effect emerged on amygdala activity. With regard to maternal or infant responsiveness, the interactions failed to reach FWE significance in the amygdala (all p_uncorrected_ > 0.004).

### Sensitivity analyses

To ensure that the interaction was not confounded by an adverse family environment, the interaction was additionally adjusted for psychosocial adversity until the age of 11 years, which did not change the results [*t*(165) = 3.96, *P*_FWE_ = 0.002]. This was also true when adjusting for adversity occurring after the age of 11 years until the 25-year assessment [*t*(165) = 3.78, *P*_FWE_ = 0.004].

Likewise, the results remained significant after controlling for lifetime internalizing psychopathology in the offspring [*t*(165) = 3.86, *P*_FWE_ = 0.003] and lifetime substance abuse [*t*(159) = 3.69, *P*_FWE_ = 0.006]. In addition, the results remained unchanged after the inclusion of absolute translation and rotation [*t*(164) = 3.98, *P*_FWE_ = 0.002].

In addition, in order to disentangle the genetic confounding of the predictors, the effect of familial risk on maternal stimulation was regressed out, and the interaction remained significant with regard to ADHD (β = ‒0.38, SE = 0.14, *P* = 0.005) and with regard to amygdala habituation [*t*(166) = 3.44, *P*_FWE_ = 0.01].

Given the nature of the caretaker analyses, analyses were rerun to consider maternal (*n* = 64 diagnoses) and paternal psychopathology (*n* = 43 diagnoses) separately. Critically, while the interaction between paternal diagnoses and maternal stimulation was not significant although in the same direction [*t*(162) = 2.15, *P*_FWE_ = 0.23], the interaction between maternal diagnoses and maternal stimulation predicted the level of amygdala habituation [*t*(166) = 3.50, *P*_FWE_ = 0.01].

### Association with child and adolescent psychopathology

Interestingly, the lifetime ADHD diagnoses were weakly associated with amygdala habituation (β = 0.18, SE = 0.08, *P* = 0.03; *R*^2^ change = 0.03, Supplementary Figure S2), indicating less activation decrease in individuals with previous ADHD. This was still significant when controlling for initial amygdala values (β = 0.17, SE = 0.08, *P* = 0.03). In contrast, amygdala habituation was unrelated to internalizing diagnoses (*P* = 0.98).

Given the reported interaction effect between familial risk and maternal stimulation on ADHD diagnoses (Holz *et al.*, 2018a), mediation of the relation between maternal stimulation and amygdala habituation by lifetime ADHD was tested, but was not significant (*P* = 0.25).

## Discussion

The present study investigated whether early maternal care and parental psychopathology interact to predict amygdala habituation. In line with our hypothesis, more maternal stimulation was related to a greater response decrement in the amygdala over time in those with high familial risk, while no such relationship emerged in the no-risk group. Further, this finding was independent of the initial level of amygdala activity and could not be attributed to a decline in attention as seen in the behavioral performance. Moreover, a greater response decrement in the amygdala was found to be related to less lifetime ADHD.

In analogy to the interaction patterns reported by our group during reward processing (Holz *et al.*, 2018a), the results of the present study reveal a similar impact of maternal care on a favorable neural response, with increased amygdala habituation in the risk group. This indicates that maternal care and the neural substrates of hot affective processing, i.e. reward and emotion might represent cross-system level protective factors, possibly alleviating the risk for externalizing disorders such as ADHD in the face of adversity. Notably, the effect patterns provide a biological basis for the protective factor model of resilience (Garmezy *et al.*, 1984), which is commonly seen on a behavioral level, with the dampening effect of maternal care being visible in the risk group only. In this vein, our results are in line with the hypothesis that early maternal care (and maternal stimulation in particular) could promote resilience to neurodevelopmental disorders in high-risk children, expressed by increased amygdala habituation. Specifically, our findings suggest that maternal psychopathology and poor maternal stimulation represent a crucial risk pathway for the development of psychopathology in the offspring, and that preventive strategies targeting early maternal care could consequently be more promising in children who are at high risk due to their mothers’ psychopathology compared to children whose fathers suffer from mental disorders. However, further research is required to investigate whether maternal *vs* paternal psychopathology contribute to children’s development through different neurodevelopmental pathways.

Numerous studies have demonstrated the importance of parental psychopathology for the development of children’s mental health (Wille *et al.*, 2008; Dean *et al.*, 2010; Van Santvoort *et al.*, 2015a). In this respect, parental psychopathology constitutes a risk factor encompassing genetic and environmental risks. While the latter entails adverse family environments, the former could be attributed to genetic liability for arousal or affect dysregulation embedding a latent transdiagnostic risk phenotype for externalizing as well as internalizing disorders (Shadur and Lejuez, 2015; McLaughlin, 2016; Weissman *et al.*, 2019; Lynch *et al.*, 2020). Although the core symptom manifestations of ADHD, CD and depression differ, they all share emotional and behavioral dysregulation within the respective symptom dimensions. Interestingly, our results suggest that this transdiagnostic risk might be buffered by maternal stimulation with regard to ADHD.

Amygdala habituation can be considered as an important neural process related to stress adaptation. Indeed, the capacity of the brain to maintain adaptive levels of arousal to predictable social stimuli might allow a more flexible adjustment to stressful situational demands, which, in turn, might foster the development of crucial socioemotional skills. In support of this, reduced amygdala habituation has already been related to disorders with disrupted social functioning (Kleinhans *et al.*, 2009; Blackford *et al.*, 2013; Swartz *et al.*, 2013; Wiggins *et al.*, 2014) and social fearfulness (Avery and Blackford, 2016), possibly indicating a deficit during social interactions. In analogy to our results, recent research has emphasized that neuroflexibility, i.e. dynamic rather than stable activity of neural substrates involved in social stress processing, might be crucial for resilient coping (Sinha *et al.*, 2016). Here, we propose that decreasing amygdala over time as a function of maternal care in individuals at familial risk might play a role in rendering them resilient to social adversity.

Neurodevelopmental studies investigating the influence of early parental care on amygdala development indicated that the amygdala represents a structure that is sensitive to maternal care and undergoes dynamic changes particularly during early life (Tottenham and Sheridan, 2009; Lupien *et al.*, 2011; Gilmore *et al.*, 2012; Fareri and Tottenham, 2016). Moreover, given its central role in the processing and production of emotional and social behavior, the amygdala is a crucial candidate to be related to the development of both internalizing and externalizing disorders. As such, there is a literature indicating that poor care from mothers suffering from depressive symptoms is associated with an acceleration of amygdala development in the offspring (Lupien *et al.*, 2011). Interestingly, this is in line with data from orphaned children, in whom the absence of the mother had similar effects on the amygdala (Mehta *et al.*, 2009; Tottenham *et al.*, 2010). However, it remains unclear which component of early caregiving needs to be focused on in terms of preventive strategies. Moreover, it should be considered that a low quality of early parental care presumably represents only one factor among others that could contribute to atypical amygdala development at an early stage. Posner and colleagues for example found in utero exposure to maternal depression to be associated with atypical amygdala-prefrontal connectivity in infants (Posner *et al.*, 2016), suggesting that further (patho-)mechanisms affecting amygdala development may even start prenatally. A deeper understanding of the various pathways, furthered by a broader intergenerational view, could help to find new approaches in prevention [also see (Duarte *et al.*, 2020)].

In our study, we focused on the relevance of early maternal care and investigated early mother–child interaction by rating maternal stimulation and responsiveness in a standardized nursing and play situation. Our results indicate a more prominent role of maternal stimulation compared to that of maternal responsiveness under conditions of high familial risk. Nevertheless, this does not necessarily imply a negligible role of maternal responsiveness, which itself has been shown to impact the emergence of disruptive behavior (Wakschlag and Hans, 1999). Rather than attributing importance solely to maternal interactive stimulation, it should be kept in mind that maternal responsiveness also contributes to the development of the child’s socioemotional well-being. However, the concept of ‘the more the better’ may not always be the case. As seen in our results on main effects, high maternal responsiveness was related to decreased amygdala habituation over time. This may seem counterintuitive given the current literature suggesting that higher maternal responsiveness is linked to better outcomes, such as lower levels of fear in the offspring (Gartstein *et al.*, 2017). Conversely, it also has to be noted that high contingencies between infants and mothers have been related to communicative stress, avoidant attachment and lower resilience (for a review, see Beebe *et al.*, 2008). As such, being less responsive could lead to increased coping abilities in the infant (Van Ijzendoorn and Hubbard, 2000). Likewise, regarding maternal stimulation, the concept of ‘the more the better’ only applied to those with high familial risk and was not true for the low-risk group, which is in line with previous evidence (Wakschlag and Hans, 1999; Holz *et al.*, 2018a).

The association between internalizing psychopathology and amygdala habituation was not replicated in the present study, which echoes previous inconsistent results regarding this relationship. While Hare *et al.* (2008) showed that individuals with higher trait anxiety failed to habituate, the opposite was found in patients with social anxiety disorder (Sladky *et al.*, 2012). Moreover, null results were obtained in patients with an internalizing disorder (Van Den Bulk *et al.*, 2016) and in typically developing controls and Autism Spectrum Disorder (ASD) patients (Swartz *et al.*, 2013). Interestingly, recent evidence suggests that the level of PTSD symptoms may not be predicted by amygdala habituation but by anterior cingulate cortex habituation, underlining that sustained activation of top-down control areas might contribute to increased arousal over time in internalizing disorders (Stevens *et al.*, 2017).

The present study investigated a linear within-subject contrast of amygdala habituation in accordance with Plichta *et al.* (2014). Most studies focused on the contrast between the first run and a later run (such as done previously; Stevens *et al.*, 2017), without taking into account the course of amygdala habituation over time. Interestingly, the pattern of amygdala habituation over time shows that the amygdala activity decreased most during the last two blocks, where the difference to the first block was significant. Therefore, future studies on amygdala habituation need to include a sufficient number of blocks in order to increase their power to detect differences in the amygdala response decrement. Notably, our results were independent of the initial amygdala activation, which is crucial given that an initially high signal might also have more opportunity to attenuate over time. Interestingly, accuracy was stable over time, which might imply that amygdala habituation does not simply represent a decrement of attention.

While this study has several strengths, such as its prospective long-term character, reaching from infancy to adulthood, some limitations should be noted. First, the study sample was enriched with children born at risk, which might have led to an underrepresentation of highly stimulating and responsive mothers. However, given the direction of the results, the interaction patterns are expected to be even stronger when including mothers, presumably in the familial risk group, who show very high levels of early care. Second, although the mother–child interaction was only considered during infancy, this parenting behavior is considered to be stable (Feldman, 2010). Third, we only found a weak inverse correlation between amygdala habituation and lifetime ADHD, which would not survive the Bonferroni correction for multiple comparisons for the two tests regarding externalizing and internalizing diagnoses performed (*P* = 0.05/2). Although this relationship may seem plausible, it has to be acknowledged that due to the lack of current diagnoses, former ADHD diagnoses were taken into account. Thus, we speculate that less amygdala habituation in those with previous lifetime ADHD may be a persisting latent vulnerability phenotype. Therefore, future studies should test amygdala habituation in patients fulfilling an ADHD diagnosis. Fourth, in the present study, we investigated the role of parental psychopathology and maternal stimulation, which are likely to be intertwined with the presence of other risk factors, such as a lower level of self-regulation and a lower socioeconomic status (McLaughlin *et al.*, 2014; McLaughlin, 2016). Furthermore, we did not assess other relevant parenting aspects such as parenting styles and prenatal exposure to psychotropic medication, which have also been shown to exert a neurodevelopmental influence (Lugo-Candelas *et al.*, 2018). Further, research is required to disentangle the role of other risk factors with regard to amygdala functioning. Finally, solely attributing positive effects of the mother–child interaction to amygdala habituation and reward processing (Holz *et al.*, 2018a) might represent an oversimplification given that these positive effects are likely to be transmitted by multiple channels, also including the shaping of hormone and neurotransmitter systems and, possibly, the microbiome (Curley and Champagne, 2016).

## Conclusion

The present study shows that individuals with high familial risk and high levels of maternal stimulation early in life exhibit a stronger amygdala habituation in adulthood. This may suggest an increased ability to flexibly adjust to situational demands in social interactions. Therefore, interventions targeting maternal care early in life, such as video feedback, might be beneficial for the offspring’s neurodevelopment.

## Supplementary Material

nsab059_Supp
