# Supplementary figures and images for: Early maternal care and amygdala habituation to emotional stimuli in adulthood
Source: Soc Cogn Affect Neurosci. 2021 May 8;16(10):1100–10. doi: 10.1093/scan/nsab059 (PMC8483279; doi:10.1093/scan/nsab059)

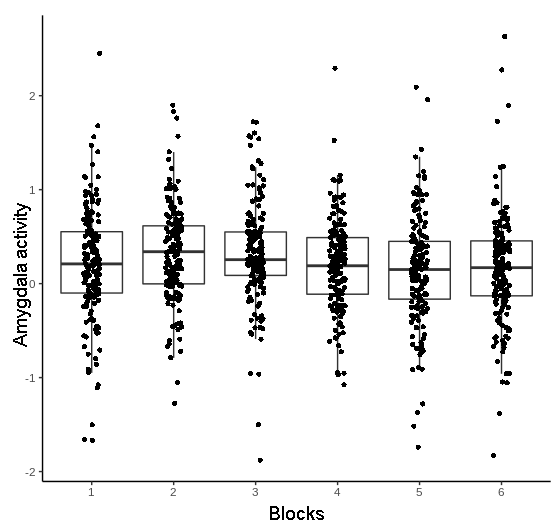

Supplement: nsab059_Supp [file nsab059_supp.zip › FigS1.tif]

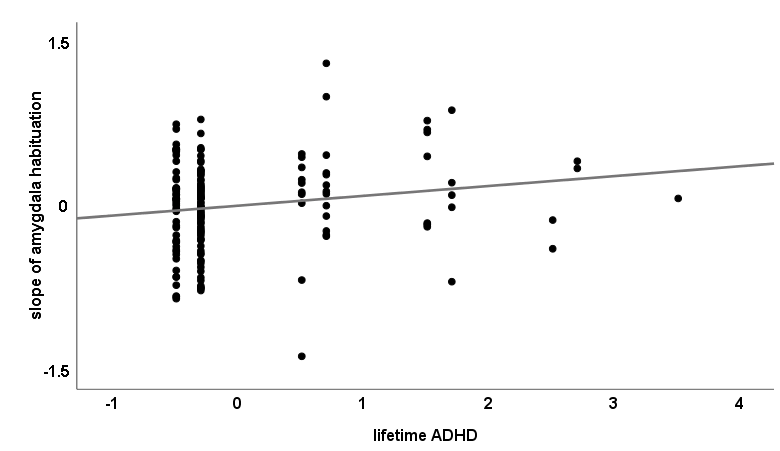

Supplement: nsab059_Supp [file nsab059_supp.zip › FigS2.tif]
