# Supplementary material for: Early maternal care and amygdala habituation to emotional stimuli in adulthood
Source: Soc Cogn Affect Neurosci. 2021 May 8;16(10):1100–10. doi: 10.1093/scan/nsab059 (PMC8483279; doi:10.1093/scan/nsab059)
Supplement: nsab059_Supp [file nsab059_supp.zip › Supplemental_information_(2).doc]

# Title page

**Early maternal care and amygdala habituation to emotional stimuli in adulthood**

Nathalie E. Holz^1^*, Ph.D., Alexander Häge^1^*, M.D., Michael M. Plichta^1,2,3^, Ph.D., Regina Boecker-Schlier^1^, Ph.D., Christine Jennen-Steinmetz^4^, Ph.D., Sarah Baumeister^1^, Ph.D., Andreas Meyer-Lindenberg^2^, M.D., Ph.D., Manfred Laucht^1,5^, Ph.D., Tobias Banaschewski^1^†, M.D., Ph.D., Daniel Brandeis^1, 5-7^†, Ph.D.

*Equal contribution; †Equal contribution

^1^Department of Child and Adolescent Psychiatry and Psychotherapy, ^2^Department of Psychiatry and Psychotherapy, ^4^Department of Biostatistics, Central Institute of Mental Health, Medical Faculty Mannheim / Heidelberg University, J5, 68159 Mannheim, Germany; ^3^Department of Psychiatry, Psychosomatic Medicine and Psychotherapy, Goethe-Universität Frankfurt am Main, Hoffmann-Str. 10, 60528 Frankfurt am Main, Germany; ^5^Department of Child and Adolescent Psychiatry and Psychotherapy, Psychiatric Hospital, University of Zurich, Neumünsterallee 9, 8032 Zurich, Switzerland, ^6^Center for Integrative Human Physiology, Winterthurerstrasse 190, 8057 Zurich, Switzerland, ^7^Neuroscience Center Zurich, University of Zurich and ETH Zurich, Winterthurerstrasse 190, 8057 Zurich, Switzerland

# Supplemental Information

**Methods** Sample, Familial Risk, Early mother-child interaction, fMRI parameters and data analysis

**References**

# Method

## Sample

The initial sample consisted of 384 children of predominantly (>99.0%) European descent, born between 1986 and 1988. Infants were recruited from two obstetric and six children’s hospitals in the Rhine-Neckar Region of Germany and were included consecutively into the sample according to a two-factorial design intended to enrich and to control the risk status of the sample (). To control for confounding effects of family environment and infant medical status, only firstborn children with singleton births and German-speaking parents were enrolled. Assessments were conducted at the age of 3 months and at regular intervals throughout development. Functional and structural magnetic resonance imaging was introduced in the most recent assessment at age 25 years. From the initial sample of 384 participants, 18 (4.7%) were excluded due to severe disabilities, and 57 (14.8%) were dropouts, leaving a final sample of 309 for the 25-year assessment. Exclusion criteria were the usual contraindications for MRI (such as heart pacemaker, neurological abnormalities, history of seizures, unconsciousness or head trauma), current psychiatric disorders as assessed by the Structured Clinical Interview for DSM-IV , and psychotropic medication. One participant was excluded due to systemic lupus erythematosus, and an additional 11 subjects were excluded due to movement >2mm. From these, data on early mother-child interaction was available for N=172. In terms of attrition, the dropout sample had higher obstetric (t(380)=4.17, p<.001) and psychosocial risk (t(380)=3.18, p=.002), more parental psychiatric diagnoses (t(380)=3.00, p=.003) as well as a lower level of maternal responsiveness (t(366)=-2.67, p=.008), however, were not different with regard to maternal stimulation, sex and sum of ADHD diagnoses. A comparison between those who participated in psychiatric assessment of the last assessment wave (including those with current diagnoses) versus those selected for the fMRI measurement, showed a higher level of maternal reactivity (t(293)=-2.22, p=.03) and lower obstetric risk (t(305)=2.81, p=.007) in the fMRI sample. No differences emerged regarding sex, ADHD, maternal stimulation, psychosocial adversity and parental psychiatric diagnoses, therefore the fMRI sample is likely to be unbiased in terms of environmental hardship and most of the variables of interest of the current investigation.

## Familial risk

A substantial degree of genetic transmission of parent-child resemblance for externalizing disorders has been demonstrated , however, various other parental psychiatric disorders including mood and anxiety disorders , substance use disorders and personality disorders have also been related to child externalizing behavior. Therefore, psychiatric diagnoses in biological parents included different diagnostic categories, such as personality, affective, substance-related and addictive, somatoform and stress disorders (0=not present, 1=present). 6 participants were excluded due to missing information on the mental health of their biological parents. The numbers of missing values for paternal psychiatric disorder for the five assessment waves were 2, 2, 6, 9, 13; here only maternal mental health was considered.

## Early mother-child interaction

The validity of the early interaction paradigm and the measures derived has been demonstrated in several publications . Early mother-child interaction data was missing for 5 subjects.

## fMRI parameters and data analysis

The imaging protocol consisted of a localizer scan followed by a BOLD-sensitive T2*-weighted echoplanar imaging sequence and a structural T1-weighted sequence. For functional imaging, a total of 183 volumes with 36 slices (matrix 64x64, resolution 3.43x3.43x3 mm, repetition time=2210ms, echo time=28 ms, flip angle=90°) covering the whole brain were acquired. The slices were inclined 20° from the anterior/posterior commissure level to minimize dropout artifacts in orbitofrontal and mediotemporal regions. The first 4 fMRI volumes were discarded to allow longitudinal magnetization to reach equilibrium. Preprocessing included slice time correction of the volumes to the first slice, realignment to correct for movement artifacts, coregistration of functional and anatomical data, spatial normalization to standard MNI (Montreal Neurological Institute) space and smoothing with a Gaussian kernel of 8mm full-width-at-half-maximum (FWHM). In addition, all analyses were rerun with a smoothing kernel of 6 mm, which did not change the results.

Table S1. Percentiles of maternal stimulation for the no risk and the risk group.

| Percentiles of maternal stimulation | No risk | Risk |
| --- | --- | --- |
| 10 | -0,921076 | -1,384029 |
| 20 | -0,585814 | -1,086674 |
| 30 | -0,252520 | -0,828085 |
| 40 | -0,045823 | -0,528063 |
| 50 | 0,088738 | -0,383444 |
| 60 | 0,357110 | -0,090034 |
| 70 | 0,599520 | 0,039012 |
| 80 | 0,967265 | 0,364704 |
| 90 | 1,656903 | 1,296447 |

Table S2. Regression models.

|  | **B** | **Std. Error** | **Beta** | **T-score** | **p-value (uncorr)** |
| --- | --- | --- | --- | --- | --- |
| **Model 1** |  |  |  |  |  |
| Familial risk by maternal stimulation | -.25 | .07 | -.42 | -3.87 | <.001 |
| Familial risk | -.02 | .07 | -.02 | -.24 | .81 |
| Maternal stimulation | .09 | .05 | .21 | 1.97 | .051 |
| sex | -.01 | .06 | -.02 | -.20 | .844 |
| Child responsiveness | .01 | .03 | .03 | .34 | .73 |
| **Model 1 including predictor by covariate interactions** |  |  |  |  |  |
| Familial risk by maternal stimulation | -.25 | .065 | -.42 | -3.82 | <.001 |
| Familial risk | -.14 | .21 | -.17 | -.68 | .50 |
| Maternal stimulation | .19 | .12 | .46 | 1.61 | .11 |
| sex | -.06 | .09 | -.07 | -.69 | .49 |
| Child responsiveness | -.009 | .05 | -.02 | -.20 | .85 |
| Familial risk by sex | .08 | .13 | .17 | .64 | .53 |
| Maternal stimulation by sex | -.07 | .07 | -.28 | -1.06 | .29 |
| Familial risk by Child responsiveness | .07 | .07 | .12 | 1.02 | .31 |
| Maternal stimulation by Child responsiveness | .02 | .03 | .06 | .77 | .44 |
| **Model 2** |  |  |  |  |  |
| Familial risk by maternal responsiveness | .06 | .06 | -.008 | -.11 | 91 |
| Familial risk | -.04 | .06 | -.05 | -.63 | .53 |
| Maternal responsiveness | .02 | .05 | .06 | .50 | .62 |
| sex | -.007 | .06 | -.008 | -.11 | .91 |
| Child responsiveness | .007 | .04 | .02 | .17 | .87 |
| **Sensitivity analyses of Model 1** |  |  |  |  |  |
| **1. including initial amygdala activity** |  |  |  |  |  |
| Familial risk by maternal stimulation | -.26 | .07 | -.40 | -3.89 | <.001 |
| Familial risk | -.03 | .07 | -.03 | -.39 | .70 |
| Maternal stimulation | .09 | .05 | .20 | 1.91 | .06 |
| sex | .006 | .07 | .007 | .10 | .93 |
| Child responsiveness | .02 | .03 | .04 | .60 | .55 |
| Initial amygdala activity | -.21 | .06 | -.28 | -3.87 | <.001 |
| **2. including psychosocial adversity** |  |  |  |  |  |
| Familial risk by maternal stimulation | -.25 | .06 | -.42 | -3.96 | <.001 |
| Familial risk | -.177 | .08 | -.21 | -2.21 | .03 |
| Maternal stimulation | .09 | .05 | .21 | 1.95 | .053 |
| sex | -.004 | .06 | -.004 | -.06 | .95 |
| Child responsiveness | .02 | .03 | .04 | .5 | .58 |
| Psychosocial adversity | .06 | .02 | .30 | 3.32 | .001 |
| **3. including stressful life events** |  |  |  |  |  |
| Familial risk by maternal stimulation | -.25 | .07 | -.41 | -3.78 | <.001 |
| Familial risk | -.03 | .07 | -.03 | -.39 | .70 |
| Maternal stimulation | .09 | .05 | .20 | 1.88 | .06 |
| sex | -.02 | .07 | -.02 | -.24 | .81 |
| Child responsiveness | .01 | .03 | .03 | .45 | .65 |
| Life events (11-25years) | .02 | .01 | .13 | 1.69 | .09 |
| **4. including lifetime internalizing psychopathology** |  |  |  |  |  |
| Familial risk by maternal stimulation | -.25 | .06 | -.42 | -3.86 | <.001 |
| Familial risk | -.02 | .07 | -.02 | -.25 | .80 |
| Maternal stimulation | .09 | .05 | .21 | 1.95 | .053 |
| sex | -.01 | .07 | -.02 | -.21 | .83 |
| Child responsiveness | .01 | .03 | .03 | .34 | .73 |
| lifetime internalizing psychopathology | .003 | .04 | .006 | .08 | .94 |
| **5. including lifetime substance abuse** |  |  |  |  |  |
| Familial risk by maternal stimulation | -.23 | .06 | -.39 | -3.69 | <.001 |
| Familial risk | -.004 | .06 | .005 | .07 | .94 |
| Maternal stimulation | .07 | .04 | .16 | 1.49 | .14 |
| sex | -.02 | .07 | -.03 | -.35 | .73 |
| Child responsiveness | .02 | .03 | .05 | .66 | .51 |
| lifetime nicotine abuse | -.004 | .009 | -.04 | -.51 | .61 |
| lifetime alcohol abuse | .01 | .01 | .06 | .72 | .47 |
| lifetime cannabis abuse | -.09 | .04 | -.20 | -2.53 | .01 |
| **6. including movement** |  |  |  |  |  |
| Familial risk by maternal stimulation | -.27 | .07 | -.44 | -3.98 | <.001 |
| Familial risk | -.02 | .07 | -.02 | -.26 | .80 |
| Maternal stimulation | .10 | .05 | .23 | 2.08 | .04 |
| sex | -.02 | .06 | -.02 | -.25 | .81 |
| Child responsiveness | .009 | .03 | .02 | .28 | .78 |
| Absolute translation | .08 | .07 | .09 | 1.07 | .29 |
| Absolute rotation | -.02 | .07 | -.03 | -.28 | .78 |
| **7. controlled for shared variance, effect of familial risk on maternal stimulation was regressed out** |  |  |  |  |  |
| Familial risk by maternal stimulation | -.24 | .07 | -.37 | -3.44 | .001 |
| Familial risk | .01 | .07 | .01 | .18 | .86 |
| Maternal stimulation | .07 | .05 | .15 | 1.37 | .17 |
| sex | .001 | .07 | .001 | .02 | .99 |
| Child responsiveness | .01 | .03 | .03 | .42 | .67 |
| **8. familial risk (maternal psychopathology only)** |  |  |  |  |  |
| Familial risk by maternal stimulation | -.26 | .07 | .36 | -3.50 | .001 |
| Familial risk | -.10 | .08 | -.11 | -1.36 | .17 |
| Maternal stimulation | .07 | .05 | .15 | 1.45 | .15 |
| sex | .001 | .07 | .001 | .01 | .99 |
| Child responsiveness | .007 | .04 | .01 | .19 | .85 |
| **9. familial risk (paternal psychopathology only)** |  |  |  |  |  |
| Familial risk by maternal stimulation | -.22 | .10 | -.20 | -2.15 | .03 |
| Familial risk | .06 | .11 | .05 | .57 | .57 |
| Maternal stimulation | .02 | .05 | .04 | .42 | .68 |
| sex | .03 | .09 | .03 | .33 | .75 |
| Child responsiveness | .04 | .05 | .07 | .87 | .39 |
| **Association with child and adolescent psychopathology: ADHD** |  |  |  |  |  |
| ADHD | -.18 | .08 | -.17 | -2.18 | .03 |
| sex | -.07 | .13 | -.04 | -.57 | .57 |
| **Association with ADHD controlled for initial amygdala activity** |  |  |  |  |  |
| ADHD | -.17 | .08 | -.16 | -2.20 | .03 |
| sex | -.08 | .12 | -.05 | -.64 | .53 |
| Amygdala activity in the first block | .38 | .10 | .28 | 3.78 | <.001 |
| **Association with child and adolescent psychopathology: internalizing diagnoses** |  |  |  |  |  |
| internalizing diagnoses | -.002 | .08 | -.002 | -.02 | .98 |
| sex | -.04 | .13 | -.02 | -.29 | .77 |

Note: for all interaction models the dependent variable was the exported amygdala peak voxel derived from the second-level SPM analysis. The whole significant amygdala cluster from model 1 was the dependent variable in the regression analysis with ADHD.

# References

Basten, M. M., Althoff, R. R., Tiemeier, H., Jaddoe, V. W., Hofman, A., Hudziak, J. J., . . . van der Ende, J. (2013). The dysregulation profile in young children: empirically defined classes in the Generation R study. *Journal of the American Academy of Child and Adolescent Psychiatry, 52*(8), 841-850 e842. doi:10.1016/j.jaac.2013.05.007

Breaux, R. P., Harvey, E. A., & Lugo-Candelas, C. I. (2014). The role of parent psychopathology in the development of preschool children with behavior problems. *Journal of Clinical Child and Adolescent Psychology, 43*(5), 777-790. doi:10.1080/15374416.2013.836451

Brennan, P. A., Hammen, C., Katz, A. R., & Le Brocque, R. M. (2002). Maternal depression, paternal psychopathology, and adolescent diagnostic outcomes. *Journal of Consulting and Clinical Psychology, 70*(5), 1075-1085.

Buchmann, A. F., Kopf, D., Westphal, S., Lederbogen, F., Banaschewski, T., Esser, G., . . . Deuschle, M. (2010). Impact of early parental child-rearing behavior on young adults' cardiometabolic risk profile: a prospective study. *Psychosomatic Medicine, 72*(2), 156-162. doi:10.1097/PSY.0b013e3181c88343

Hicks, B. M., Foster, K. T., Iacono, W. G., & McGue, M. (2013). Genetic and environmental influences on the familial transmission of externalizing disorders in adoptive and twin offspring. *JAMA Psychiatry, 70*(10), 1076-1083. doi:10.1001/jamapsychiatry.2013.258

Hussong, A. M., Huang, W., Curran, P. J., Chassin, L., & Zucker, R. A. (2010). Parent alcoholism impacts the severity and timing of children's externalizing symptoms. *Journal of Abnormal Child Psychology, 38*(3), 367-380. doi:10.1007/s10802-009-9374-5

Laucht, M., Esser, G., Baving, L., Gerhold, M., Hoesch, I., Ihle, W., . . . Schmidt, M. H. (2000). Behavioral sequelae of perinatal insults and early family adversity at 8 years of age. *Journal of the American Academy of Child and Adolescent Psychiatry, 39*(10), 1229-1237. doi:10.1097/00004583-200010000-00009

Laucht, M., Esser, G., & Schmidt, M. H. (1997). Developmental outcome of infants born with biological and psychosocial risks. *Journal of Child Psychology and Psychiatry, 38*(7), 843-853.

Laucht, M., Esser, G., & Schmidt, M. H. (2001). Differential development of infants at risk for psychopathology: the moderating role of early maternal responsivity. *Developmental Medicine and Child Neurology, 43*(5), 292-300.

Marmorstein, N. R., Iacono, W. G., & McGue, M. (2009). Alcohol and illicit drug dependence among parents: associations with offspring externalizing disorders. *Psychological Medicine, 39*(1), 149-155. doi:10.1017/S0033291708003085

Nigg, J. T., & Hinshaw, S. P. (1998). Parent personality traits and psychopathology associated with antisocial behaviors in childhood attention-deficit hyperactivity disorder. *Journal of Child Psychology and Psychiatry and Allied Disciplines, 39*(2), 145-159.

Renk, K., Oliveros, A., Roddenberry, A., Klein, J., Sieger, K., Roberts, R., & Phares, V. (2007). The relationship between maternal and paternal psychological symptoms and ratings of adolescent functioning. *Journal of Adolescence, 30*(3), 467-485. doi:10.1016/j.adolescence.2006.05.001

Schmid, B., Blomeyer, D., Buchmann, A. F., Trautmann-Villalba, P., Zimmermann, U. S., Schmidt, M. H., . . . Laucht, M. (2011). Quality of early mother-child interaction associated with depressive psychopathology in the offspring: a prospective study from infancy to adulthood. *Journal of Psychiatric Research, 45*(10), 1387-1394. doi:10.1016/j.jpsychires.2011.05.010

Stepp, S. D., Whalen, D. J., Pilkonis, P. A., Hipwell, A. E., & Levine, M. D. (2012). Children of mothers with borderline personality disorder: identifying parenting behaviors as potential targets for intervention. *Personality Disorders, 3*(1), 76-91. doi:10.1037/a0023081

Wittchen, H. U., Zaudig, M., & Fydrich, T. (1997). *SKID - Strukturiertes Klinisches Interview DSM-IV Achse I und II.* Göttingen (DE): Hogrefe.
